# Inter-individual differences in muscle damage following a single bout of high-intense whole-body electromyostimulation

**DOI:** 10.3389/fspor.2024.1454630

**Published:** 2024-10-23

**Authors:** Marc Teschler, Melina Waranski, Boris Schmitz, Frank C. Mooren

**Affiliations:** ^1^ Department of Rehabilitation Sciences, Faculty of Health, University of Witten/Herdecke, Witten, Germany; ^2^ DRV Klinik Königsfeld, Center for Medical Rehabilitation, Ennepetal, Germany

**Keywords:** creatine kinase, exercise, inter-individual difference, muscle damage, myoglobin, WB-EMS

## Abstract

**Purpose:**

This brief report aimed to characterize inter-individual training responses following a single session of high-intense whole-body electromyostimulation (WB-EMS) using markers of muscle damage over a period of 72 h.

**Methods:**

Twelve healthy individuals (5 men, 7 women; 32.0 ± 7 years) participated in a single 20-minute high-intensity WB-EMS training session. Markers of muscle damage, creatine kinase (CK) and myoglobin (Mb), were assessed before and immediately after training, as well as at 1.5, 3, 24, 48 and 72 h post-exercise. Lactate levels were determined pre- and post-exercise.

**Results:**

Overall, WB-EMS induced significant CK elevations, peaking at 72 h (18.358 ± 21.380 U/L; *p* < 0.01), and correlating Mb levels peaking at 48 h (1.509 ± 1.394 ng/dl, *p* < 0.01). Despite significant inter-individual variability in CK levels, both slow (SR) and fast responders (FR) were identified. FR showed significant increases in CK at all time points post WB-EMS (*p* < 0.05), whereas CK in SR significantly elevated after 48 h. Post-WB-EMS lactate concentration was identified to predict peak CK and Mb levels (r ≥ 0.65, both *p* < 0.05).

**Conclusion:**

High-intensity WB-EMS has the potential to induce severe muscle damage, as indicated by elevated levels of CK and Mb. We identified two distinct groups of individuals, SR and FR, indicating variability in response to WB-EMS. Furthermore, we suggest that individual responses to WB-EMS can be predicted based on post-WB-EMS lactate concentration.

## Introduction

In the past decade, research on whole-body electromyostimulation (WB-EMS) has increased significantly, demonstrating its efficacy in altering body composition and enhancing performance across various health conditions and age groups (1, 2). This has led to a massive increase in WB-EMS centers and available home-training supplies, bringing WB-EMS to a large and often untrained population. Since WB-EMS application stimulates up to eight muscle groups simultaneously, the potential risks associated with too-intense and unsupervised use should not be underestimated. Excessive WB-EMS training, characterized by intense current impulses, may lead to severe muscle damage predominantly in untrained, unaccustomed users, characterized by significant increases in serum creatin kinase (CK) and myoglobin levels (3, 4).

Both CK and Mb are commonly used as markers of training intensity, influenced by various factors such as exercise type and individual physiological variations, including ethnicity and age (5). CK, a central enzyme in muscle cellular energy metabolism, facilitates the transfer of phosphate between creatine phosphate and adenosine triphosphate (ATP), providing energy, especially during short, intense exercise. Elevated blood levels of the CK-muscle type isoform, primarily found in skeletal muscle, indicate muscle damage or injury (5–7). Similarly, myoglobin (Mb), which stores and transports oxygen in muscle tissue, follows a parallel pattern. When muscle damage occurs, both CK and Mb are released into the bloodstream, marking muscle injury, typically defined as exertional rhabdomyolysis when CK levels exceeding 1,000 U/L (8). In case of Mb, it is important to consider its solubility during severe muscle damage, as Mb accumulation in the renal tubulus can lead to Mb-induced nephropathy, posing a risk to kidney health (9).

Given the current scientific focus on individual responses to (standardized) training (e.g., high vs. low responding) and sex-specific differences in physiological training adaptation (10–13), it is of particular interest to investigate the individual response to intense WB-EMS training since knowledge is still limited. Additionally, identifying markers that may indicate unfavorable outcomes is crucial. This report examines the inter-individual WB-EMS response by assessing serum CK and Mb levels over a time course of up to 72 h and analyzes potential predictors for the extent of muscle damage. Notably, we intentionally applied a non-recommended protocol for an initial WB-EMS application to provoke unusually high physiological responses, particularly in WB-EMS novices, deviating from the established guidelines for WB-EMS (14).

## Materials and methods

Twelve (5 male; 7 female) healthy and recreationally active WB-EMS novices (for characteristics see Table 1) performed one single high-intensity session of WB-EMS training using an established protocol (3). Written informed consent was obtained from all participants. The study adhered to the ethical principles of the Declaration of Helsinki and was approved by the ethics committee of University Witten/Herdecke (#91/2018). Participants had no history of cardiovascular or musculoskeletal diseases, orthopedic problems, or contraindications for WB-EMS (15).

**Table 1:** Peak values of muscle damage markers following WB-EMS by response.

Subject	Sex	Age	Height	Weight	SMM		Lactate	CK	Myoglobin	CRP
		[Years]	[cm]	[kg]	[kg]	[%]	[m/mol]	[U/L]	[mg/dl]	[mg/dl]
Fast responder (FR)										
1	Female	28	172.6	73.1	29.3	40.1	1.96	4,919^a^	515.7^a^	0.58^b^
2	Male	44	183.2	81.2	37.1	45.7	2.87	27,501^b^	3,064.9^a^	0.45^b^
5	Male	32	195.4	103	46.6	45.2	4.82	33,071^b^	2,579.3^a^	1.01^b^
9	Female	27	170.0	65.3	27.0	41.3	2.95	32,701^b^	2,608.3^a^	0.11^b^
10	Female	33	162.0	55.3	22.0	39.8	5.05	74,354^b^	7,097.4^b^	1.03^b^
	Mean	33	176.6	75.6	32.4	42.4	3.53	34,511*	3,173.1**	0.64***
	SD	7	12.9	18.1	9.6	2.8	1.34	25,088	2,405.5	0.39
Slow responder (SR)										
3	Female	29	170.8	72.3	26.2	36.2	1.79	1,398^b^	219.1^a^	0.26
4	Female	23	174.0	72.1	27.6	38.3	3.02	8,135^b^	921.2^a^	0.05
6	Female	34	176.4	82.4	30.0	36.4	1.70	585^a^	129.8^a^	0.11^b^
7	Male	33	185.0	85.5	39.2	45.8	3.58	5,331^b^	558.3^a^	0.17^b^
8	Female	42	176.0	61.9	27.0	43.6	4.31	9,898^b^	1,694.5^b^	0.07^b^
11	Male	18	169.0	74.1	34.8	47.0	2.40	20,029^b^	1,083.4^b^	0.08
12	Male	37	177.0	72.7	33.9	46.6	3.34	2,581^b^	365.4^b^	0.14^b^
	Mean	31	175.5	74.4	31.2	42.0	2.88	6,851	710.2	0.13
	SD	8	5.2	7.7	4.9	4.9	0.96	6,759	558.7	0.07
Overall MEAN		31.7	176.0	74.9	31.7	42.2	3.15	18,375	1,736.4	0.34
SD		7.4	8.7	12.3	6.8	4.0	1.13	21,369	1,970.5	0.36

Subject values are given as absolute and peak values; group values (FR, SR) are given as mean and standard deviation (SD). Statistical significance was tested using repeated measures ANOVA.

^a^ after 48 h.

^b^ after 72 h.

* *p* < 0.05.

** *p* < 0.01.

*** *p* < 0.001; SMM, skeletal muscle mass; CK, creatine kinase; CRP, C-reactive protein; peak value.

### WB-EMS session

The clinically supervised WB-EMS session took place between 7 and 9 am at the medical rehabilitation center Klinik Königsfeld, Ennepetal, Germany. Prior to the WB-EMS session and during the time course, participants were instructed to maintain their usual activity level, nutrition, and hydration status.

The CE-certified WB-EMS device and equipment (miha bodytec type-II, Gersthofen, Germany) was utilized as described (1, 16), in combination with an established load protocol (biphasic, 85 Hz frequency, 350 µs pulse width, 0.4-s pulse ramp, and a 3:2 current-rest ratio of 6 s vs. 4 s) (3). The WB-EMS session (20 min; 4 sets, 6 exercises with 5 repetitions each) was conducted dynamically, with stimulation applied primarily on top of eccentric muscle contractions, including movements such as squatting, lunge movements and combined arm movements (2). Individual current adjustments were applied to ensure muscular exhaustion, defined as rating of perceived exertion (RPE) of ≥18 via the 6–20 Borg Scale (17). Close monitoring, with a ratio of 1 trainee to 1 trainer, ensured standardized movement patterns throughout the session.

### Assessments

Anthropometric data were obtained using the seca216 (seca, Hamburg, Germany) for height measurement and a direct-segmental multi-frequency bioelectrical impedance analysis device (Inbody720, BioSpace, Seoul, Korea) for weight, skeletal muscle mass (SMM), and extracellular water (ECW) assessment, serving as indicator for training-induced plasma volume shifts. Blood samples were collected from the antecubital vein pre and immediately post WB-EMS, and at five additional time points (after 1.5, 3, 24, 48, and 72 h), and analyzed at SYNLAB MVZ Laboratory GmbH (Leverkusen, Germany) for CK, Mb, and C-reactive protein (CRP). To monitor muscle damage-induced rhabdomyolysis, participants were instructed to promptly report changes in urine color during the timespan of 72 h. Lactate concentration was measured using capillary pre and post blood samples taken from participants’ earlobes (20 μl heparinized capillary) using the Biosen S-line automated analyzer (EKF Diagnostics, Magdeburg, Germany). Muscle exertion was assessed through maximal isometric leg strength testing (extension and flexion) pre and post WB-EMS using DIERs myoline professional device (DIERs Biomedical Solutions, Schlangenbad, Germany).

### Statistical analysis

Statistical analyses were conducted using SPSSv23 software (IBM, Armonk, NY, USA) and Prism 9.2 (GraphPad Software, La Jolla, CA, USA). Data are presented as mean ± standard deviation (SD). Statistical significance was set at *p* < 0.05. Non-normal distribution was assessed using Kolmogorow-Smirnov Test. Differences from baseline and differences between responding groups were analyzed using repeated measures ANOVA. Correlation analysis of peak values was performed using the bivariate Spearman correlation coefficient (*r*).

## Results

Participants completed the protocol at a RPE of 18.3 ± 1.0, resulting in muscle fatigue, as evidenced by a reduced maximal isometric strength (leg extension: −145.8 ± 64.3 *N*, *p* < 0.001; leg flexion: −41.8 ± 52.8 *N*, *p* < 0.05). The protocol was performed under aerobic conditions, with a mean post-exercise lactate concentration of 3.15 ± 1.13 mmol/L (*p* < 0.05). ECW remained constant after WB-EMS (− 0.06 ± 0.27 L; *p* = 0.072), indicating no significant shift in plasma volume.

Immediately post-exercise, initial signs of muscle damage were identified through a significant increase in overall CK (pre-exercise, 178 ± 192 vs. post-exercise, 198 ± 201 U/L; *p* < 0.001) and Mb concentrations (pre-exercise, 38 ± 12 vs. post-exercise, 204 ± 183 ng/dl; *p* < 0.05). Subsequently, WB-EMS induced substantial and exponential elevations in both mean CK levels (at 1.5 h: 302 ± 219; 3 h: 486 ± 341; 24 h: 3,320 ± 3,421; 48 h: 10,584 ± 10,865; and 72 h: 18,375 ± 21,369 U/L) and Mb levels (at 1.5 h: 482 ± 479; 3 h: 457 ± 572; 24 h: 379 ± 347; 48 h: 1,510 ± 1,394; and 72 h: 1,320 ± 1,924 ng/dl). Peak levels were predominantly observed after 48 h for Mb and after 72 h for CK (all *p* < 0.05); with a strong correlation between CK and Mb concentrations (*r* = 0.977; *p* < 0.001).

**Figure 1:**
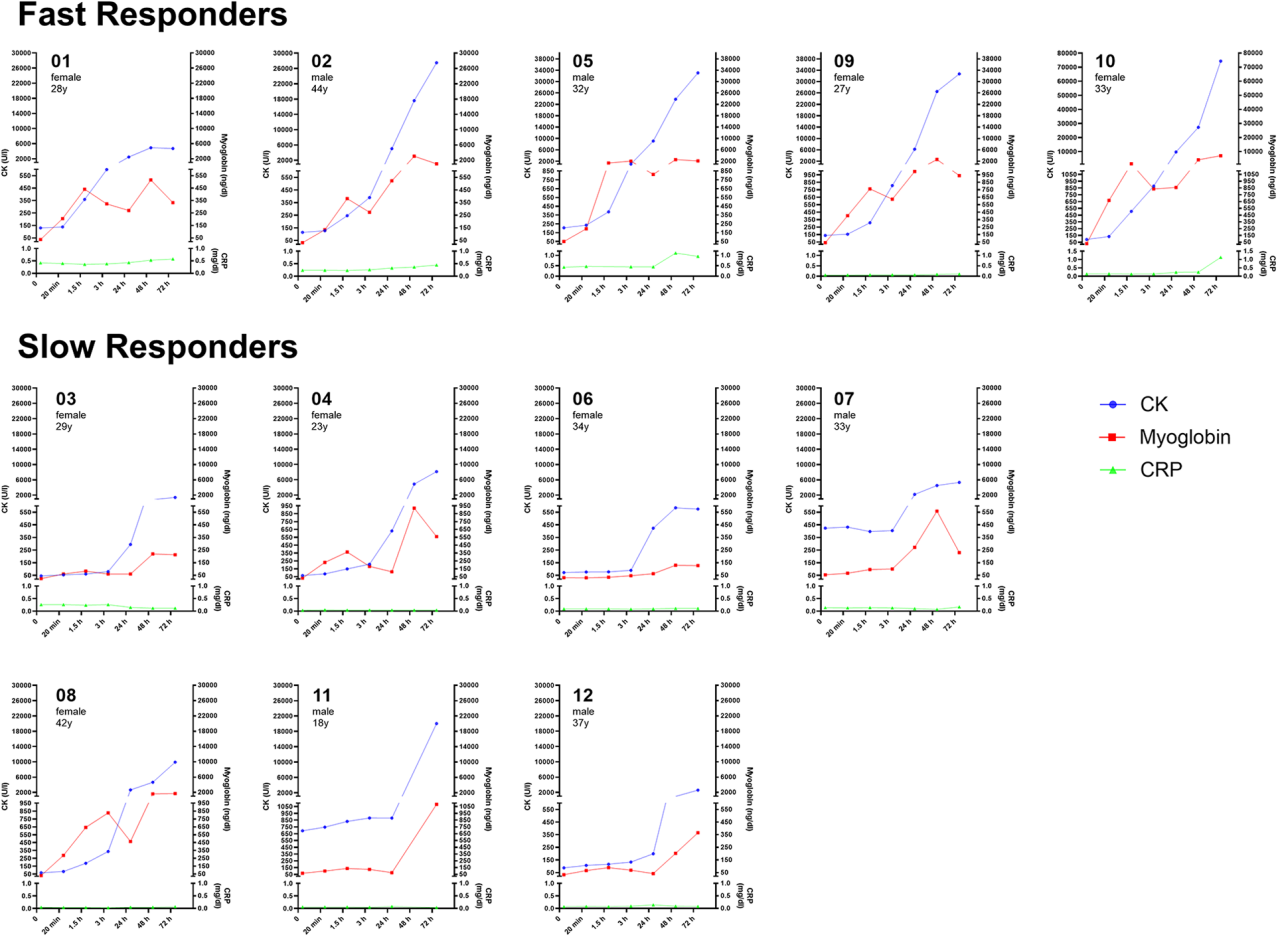
Individual time course of muscle damage markers (creatin kinase, myoglobin) and acute inflammation marker C-reactive protein by responder category.

Substantial inter-individual variability was evident regarding the maximal levels in both muscle damage markers, ranging from 585 to 74,354 U/L for CK and 130–7,097 ng/dl for Mb (see Table 1 and Figure 1).

Upon closer examination of individual time courses over time, two distinct profiles were identified, categorized as slow responders (SR) and fast responders (FR). A comparison between both groups revealed significant differences for CK at 3 h (*p* = 0.019), 24 h (*p* = 0.013), and 48 h (*p* = 0.013), and Mb after 1.5 h (*p* = 0.035), 24 h (*p* = 0.011), and 48 h (*p* = 0.025), with FR exhibiting higher peak levels for CK (34,511 ± 25,088 U/L) compared to SR (6,851 ± 6,759 U/L; *p* = 0.018) and for Mb (FR: 3,173 ± 2,406 vs. SR: 710 ± 559 ng/dl; *p* = 0.009). Additionally, CRP showed a highly significant time × group effect at 72 h, with elevations only in the FR group (*p* < 0.001).

**Figure 2:**
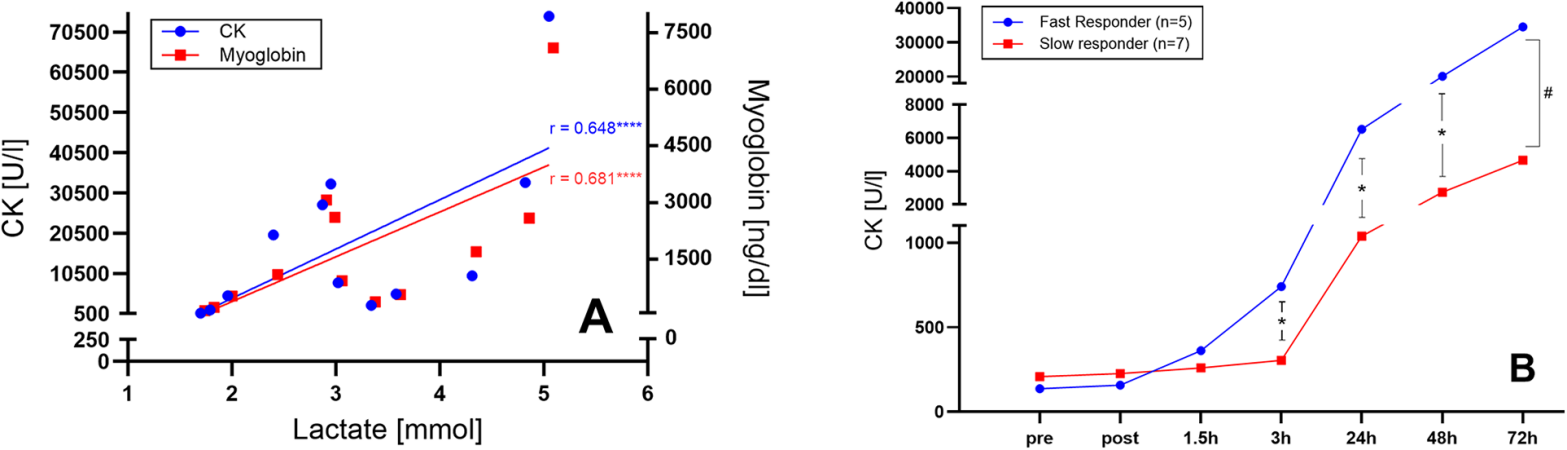
**(A)** Post-WB-EMS lactate levels predict muscle damage. Correlation and linear regression of peak creatine kinase (CK) and myoglobin levels with post-WB-EMS lactate concentration. Individual data points are shown. ****, significant correlation using bivariate Spearman correlation coefficient (*r*). **(B)** Time-course of CK levels identifies two distinct WB-EMS responder profiles. Mean CK values at the respective time points are shown. Curves were modelled using nonlinear fourth order polynomial regression. #, significant between-group interaction effect using two-way repeated-measures ANOVA, *p* < 0.0001. *, significantly different at time point, *p* < 0.05.

Of importance, individual characteristics such as sex, age, SMM, or RPE did not affect maximal levels of CK and Mb. Notably, post-WB-EMS lactate levels exhibited a strong correlation with subsequent peak levels of both muscle damage markers, suggesting predictive potential for an individual WB-EMS response (CK: *r* = 0.648, *p* = 0.02; Mb: *r* = 0.681, *p* = 0.01; Figure 2).

## Discussion

This report aimed to highlight the inter-individual variability in muscle damage induced by WB-EMS and to identify potential predictive factors for long-term CK and Mb elevations. A key finding is the identification of two distinct inter-individual response profiles to WB-EMS, with the extent of muscle damage potentially predictable by measuring post-WB-EMS lactate concentrations.

In general, markers of muscle damage exhibit heterogeneous increases depending on the type, extent, and intensity of exercise. For instance, CK levels can range from approximately 1,000 U/L after conventional resistance exercise to as high as 80,000 U/L following exhaustive eccentric exercise. While established laboratory reference values for CK typically fall between 60 and 400 U/L, various thresholds have been defined to categorize responses to exercise: low responders defined as <500 U/L, medium responders between 500 and 2,000 U/L, and high responders as >2,000 U/L (18). However, WB-EMS is known to substantially increase CK and Mb levels—by as much as 100-fold and 40-fold, respectively (3, 4)—when specific quality criteria are not met (15). This suggests that conventional response categories may not be entirely appropriate for WB-EMS application.

The current report not only provides further evidence that low and high responders to WB-EMS exist in terms of muscle damage, but our observation revealed two distinct groups based on time-dependent CK increases. The FR (fast responder) group exhibited a direct increase of CK and Mb immediately after WB-EMS (subjects 01, 02, 05, 09, 10; see Figure 1), suggesting a heightened sensitivity or reactivity to the WB-EMS stimulus. In contrast, the SR (slow responder) group demonstrated a delayed progression, with serum concentrations remaining constant for up to 24 h and showing ∼80% lower peak levels (subjects 03, 04, 06, 07, 08, 11, 12; see Figure 1). These temporal patterns may depend on various factors, including genetic and epigenetic factors, fiber type composition, and environmental or behavioral aspects (6, 19, 20). Notably, CK and Mb levels in our study appeared to be independent of sex, age and SMM.

In addition, we found that post-exercise lactate concentrations could serve as a predictor of the subsequent development of muscle damage markers. Thus, measuring capillary lactate levels during and immediately after an initial WB-EMS session may serve as valuable tool to (1) prevent excessive WB-EMS intensity, (2) provide individualized post-WB-EMS information on potential development of muscle damage, and (3) tailor personalized recovery strategies before the next WB-EMS session.

### Perspectives and significance

The inter-individual differences in muscle damage response highlight the importance of tailored training regimes. With two distinct response profiles—slow and fast responders—post WB-EMS lactate levels have shown promise as a predictive indicator of muscle damage. Routine monitoring of lactate concentration during and/or after WB-EMS may provide a simple, cost-effective method to adjust training intensity, particularly for newcomers. This monitoring approach ensures tailored recovery periods and regeneration strategies and minimizes the risk of injury from overuse. Further research should investigate whether acute lactate concentrations, comparable to CK levels, decrease over time with repeated WB-EMS sessions, potentially indicating improved muscle adaptation and tolerance to strain.

## Data Availability

The original contributions presented in the study are included in the article/Supplementary Material, further inquiries can be directed to the corresponding authors.
